# U.S. transgender women’s preferences for microeconomic interventions to address structural determinants of HIV vulnerability: a qualitative assessment

**DOI:** 10.1186/s12889-021-11471-8

**Published:** 2021-07-14

**Authors:** Tonia Poteat, Larissa Jennings Mayo-Wilson, Nastacia Pereira, Brittanni N. Wright, Shelby A. Smout, Ashlee N. Sawyer, Lauretta Cathers, Rick S. Zimmerman, Sheila R. Grigsby, Eric G. Benotsch

**Affiliations:** 1 Department of Social Medicine, University of North Carolina at Chapel Hill School of Medicine, CB #7240, Chapel Hill, NC 27516 USA; 2 Department of Applied Health Sciences, Indiana University School of Public Health, 1025 E. 7th Street, Bloomington, IN 47405 USA; 3 Global & Public Health Division, Johns Hopkins University School of Nursing, Community, 525 N. Wolfe Street, Baltimore, MD 21205 USA; 4 Department of Psychology, Virginia Commonwealth University, 806 West Franklin Street, Richmond, VA 23284 USA; 5 Wayne State University, College of Nursing, 5557 Cass Avenue, Detroit, MI 48202 USA; 6 University of Missouri St. Louis, College of Nursing, 221 NAB South Campus, University Blvd, St. Loius, MO 63121 USA

**Keywords:** Transgender women, Housing, Structural, Intervention development, Employment, Economic, Qualitative, U.S., HIV, Gender, Minority

## Abstract

**Background:**

Transgender women in the United States (U.S.) experience a disproportionate burden of HIV infection and challenges to engagement in HIV prevention and care. This excess burden is driven by structural and economic inequities. Microeconomic interventions may be effective strategies for reducing HIV inequities for this population. However, few studies have explored transgender women’s preferences for microeconomic interventions to address structural determinants of HIV vulnerability.

**Methods:**

We conducted individual interviews with 19 adult transgender women in 2 U.S. cities (Richmond, VA and St. Louis, MO) who reported one or more sexual risk behaviors and recent economic hardship related to employment/income, housing, or food security. Interviews were recorded, transcribed, and analyzed using thematic content analysis.

**Results:**

The majority (74%) of transgender women were racial/ethnic minorities with mean age of 26.3 years. 89% were currently economically vulnerable; and 23% were employed full-time. 37% reported living with HIV. Participants expressed strong support for unrestricted vouchers, with many expressing the need for funds to support gender-affirming interventions. Assistance with how to budget and save and support for job acquisition, career planning, and employment sustainment were also preferred, including access to non-stigmatizing employment. Visible transgender leadership, group empowerment, and small (rather than large) numbers of participants were considered important aspects of intervention design for transgender women, including outreach through existing transgender networks to facilitate inclusion. Incorporating HIV counseling and testing to reduce vulnerability to HIV was acceptable. However, transgender women enrolled in the study preferred that HIV not be the focus of an intervention.

**Conclusions:**

Flexible microeconomic interventions that support gender affirming interventions, improve financial literacy, and provide living-wage non-stigmatizing employment are desired by economically vulnerable transgender women. While not focused on HIV, such interventions have the potential to reduce the structural drivers of HIV vulnerability among transgender women.

## Background

Transgender women in the United States face well-documented inequities in HIV prevalence, uptake of HIV pre-exposure prophylaxis, and HIV viral suppression [1–5]. According to a 2018 meta-analysis of HIV among the U.S. transgender population conducted by the Centers for Disease Control and Prevention (CDC), laboratory-confirmed HIV prevalence in transgender women is estimated to be 14.1%, more than 45 times the prevalence in the U.S. population (0.3%) [6, 7]. HIV prevalence is even higher among Black and Latina transgender women, with approximately 44% of Black transgender women and 25.8% of Latina transgender women living with HIV, compared to 6.7% of White transgender women [6].

Multiple studies have highlighted the structural and economic drivers of HIV vulnerability among transgender women, including income insecurity [8–10], health care costs [8, 11], housing instability [8–10, 12–15], and un- and underemployment [10, 13, 14]. The unemployment rate among transgender people of color in 2015 (20%) was four times higher than the national average (5%) [16], contributing to 29% of U.S. transgender women living in poverty (<$14,000 annual income) [16]. Approximately one-third (30%) of U.S. transgender women also report having experienced homelessness in their lifetime [16]. Prior qualitative research suggests that initiatives to promote economic stability and access to safe gender-affirming medical care can play an important role in reducing HIV vulnerability among transgender women [17].

Given the persistent association of these structural factors with HIV, there has been growing recognition of the importance of interventions that address the social and economic forces that underpin HIV vulnerability [18]. With the notable exception of the Transgender Women of Color Special Project of National Significance (SPNS) funded by the U.S. Health Resources and Services administration from 2012 to 2017 [19], most HIV prevention and care interventions with transgender women in the U.S. have been behavioral [20]. Interventions that address the significant economic challenges faced by transgender women are sorely needed. Developing such programs requires understanding the needs and priorities of economically vulnerable transgender women.

We found only one study of microeconomic interventions with transgender women in the published literature. Lall et al. included cisgender and transgender women in a qualitative study of the acceptability of microeconomic interventions among sex workers in Malaysia [21]. The study found that participants were generally supportive of the proposed interventions designed to develop alternatives to sex work; however, they did note transphobia as a significant barrier to employment for transgender participants. Lall et al. did not broadly explore types of interventions that transgender women, in particular, would find useful as their focus was solely on how to reduce engagement in sex work.

In this study, we sought to understand the specific perspective of U.S. transgender women on microeconomic interventions, regardless of their current income-generating activities. We also sought to explore the acceptability of various types of microeconomic interventions and how participants’ felt those interventions should be structured.

## Methods

### Study design

We used a cross-sectional, qualitative research design. In-depth interviews with adult transgender women living in 2 U.S. cities, St. Louis, Missouri and Richmond, Virginia were conducted from July 2018 to March 2019.

### Setting

According to the 2018 HIV Surveillance Report, Richmond and St. Louis ranked 21st and 64th, respectively, in number of HIV diagnoses in U.S. metropolitan statistical areas of residence [7]. However, despite the disproportionate burden of HIV among transgender women nationally, there were no disaggregated HIV surveillance data for transgender women in either area. A previous non-probability study of HIV among transgender women in the mid-Atlantic region found that 32% were living with HIV and 10% reported their HIV status as unknown [22].

Statewide data from the most recent U.S. Transgender Survey [16] indicate that 23% of transgender people in Virginia were living in poverty, 26% had experienced homelessness, 6% were unemployed, and 66% lacked gender congruent identity documents (23% because they could not afford it) [23]. In Missouri, 27% of transgender people were living in poverty, 31% had experienced homelessness, 19% were unemployed, and 76% lacked gender congruent identity documents (36% because they could not afford it) [24]. Data from transgender women in Richmond indicate that over a third (39%) were unemployed, 52% reported housing instability in the prior year, and 62% reported annual income <$15,000 [22, 25].

### Eligibility and recruitment

Eligibility criteria and recruitment methods have been described in detail elsewhere and are outlined below [17]. Eligible participants included transgender women aged 18 to 50 years who were: (a) previously or currently economically vulnerable, defined as being unemployed, employed < 19 h per week, low income (< Medicaid poverty threshold), lacking a place to stay in the last 12 months (e.g., housed by shelter, streets, hotel, or friends), or missing a meal due to lack of food or money in the last 12 months; and (b) previously or currently behaviorally at-risk for HIV, defined as reporting condomless sex in the past 6 months plus an additional HIV risk factor in the past 6 months, such as transactional sex, sex while using alcohol or drugs, multiple sex partners, or one-time sex partners. Eligibility was determined by self-report using a computer-administered screening tool. The screening tool obtained information on age, sex assigned at birth, current gender identity, current city residence, employment status, housing and food insecurity, and recent sexual behaviors. Information on education, race, ethnicity, HIV status, access to HIV information and care, sexual partners, and hormonal therapy use was also obtained in order to characterize the transition and sexual health context of enrolled transgender women.

All transgender women were recruited using either in-person or online strategies [17]. A recruitment flyer was emailed to, posted, and distributed on-site in each city at community-based organizations (CBOs) that provide services to transgender women, an HIV clinic, and other venues and events frequented by transgender women. The flyers described the purpose of the study, eligibility criteria, planned activities, and contact information for the study team. Participating site managers were also asked to email flyers to potential participants and to refer by word-of-mouth any potentially-eligible transgender women. This process enabled the study to recruit individuals who were more likely to meet the eligibility criteria. In both cities, a member of the study team then visited each site on scheduled recruitment days to introduce potentially-eligible transgender women to the study. Interested transgender women were screened and, if eligible, completed informed consent. Those who consented completed the qualitative interview.

For online recruitment, we described the purpose of the research on a study-managed Facebook page [17]. We invited any potentially-eligible transgender women to complete an online screening tool via a secure web link. This web link was also circulated by email via the CBO listservs. Transgender women who completed the online screening were then invited to provide their name and contact information for scheduling. Those who met the eligibility criteria were contacted by the study team to schedule an in-person interview at a mutually agreeable location, such as the CBO or a university campus meeting room near the study office. Reminder text messages, emails, or phone calls were sent to participants whose interviews were scheduled more than 1 day later. All transgender women who completed the screening tool (regardless of eligibility status) received payment for their time, $5 in cash or via an electronic gift card in Richmond and $10 via an electronic gift card in St. Louis. Incentives varied slightly between the two study sites in response to community preferences regarding the amount and type of incentive offered.

### Data collection

Details of data collection have been described elsewhere and are outlined below [17]. The racially diverse research team was comprised of cisgender women and men. Qualitative interviews were conducted by a team of study investigators and four RAs: two RAs in St. Louis and two RAs in Richmond who used a semi-structured interview guide with open-ended questions. All RAs had previous qualitative research experience and received an additional five-hour training with mock interviews and role plays, in which they reviewed the study protocol and interview guide, interviewing techniques, human subjects’ requirements, and information on the local transgender community. Study investigators developed the interview guide with contributions from each of the two community advisory boards (CABs) in St. Louis and Richmond. The CABs included transgender women, transgender men, and cisgender persons who were members of local governmental or CBOs that provided medical, social, and/or mental health services to transgender individuals and/or persons living with HIV.

Following review by the CABs, the final interview guide asked enrolled transgender women to provide feedback on potential HIV and microeconomic interventions that were tailored to U.S. transgender women. Specific topics included in the interview guide were as follows: employment readiness programs, links to supportive economic services, personal finance education, gender transition vouchers, and the structure of integrated HIV prevention. We also asked participants to describe any other potential microeconomic interventions that would be helpful for transgender women, how they felt about small group structure for future interventions, and their suggestions for the best way to recruit transgender women for a microeconomic intervention. Each topic was discussed in detail. The interview guide also included questions on economic hardship and gender affirmation, and those data have been previously analyzed and published [17]. All interviews were conducted in English. They lasted approximately 60 to 90 min and were audio-recorded. All interviewed participants were given $50 in cash immediately after the interview.

### Sample size

The target sample size was 20 transgender women: 10 in St. Louis and 10 in Richmond. A small qualitative sample was chosen given the exploratory aims of the study. To achieve saturation with a relatively small sample, we used a purposive homogeneous sampling strategy of transgender women who were currently or previously experiencing economic hardship and recent sexual risk-taking. Homogeneous sampling strategies select a sub-group of individuals with common circumstances in order to yield a more focused analysis [26, 27]. We also used a prolonged interviewing period (up to 90 min) with detailed questions to elicit in-depth perspective from participants [28].

The final sample size was 19 transgender women. Participant recruitment was ended at a sample size equal to 19 participants due to reaching information saturation, where no new information was obtained. The wealth of recommendations and preferences provided by participants increased our confidence that saturation was met at both sites. It was equally important to minimize potential research burden and recruitment fatigue among transgender women.

### Analysis

All interviews were transcribed verbatim and included in the analysis. We used an integrated content analysis methodology to categorize transcribed text based on emergent themes within previously identified topics [29, 30] related to planning for a future microeconomic intervention. The previously identified topics corresponded to the questions asked in the interview guide. We then querried the data to identify emergent patterns and responses within each topic. To achieve this, two co-authors (a co-investigator and a trained graduate RA) developed a preliminary codebook based on a close reading of the 19 transcripts, and coding test of three transcripts (~ 15%). Comments and edits to the preliminary codebook were then obtained from the study investigators based on readings of the transcripts and discussions of early findings.

Next, two co-authors used a finalized codebook to code transcripts over the period of July to September 2020 using ATLAS.ti, a qualitative data analysis software (Version 8.3.1, https://atlasti.com, Corvallis, OR, USA). During the process of coding the interview text, coders developed analytical memos – noting potential themes, patterns, interpretations, and relationships between codes. Notes developed by coders were then reviewed and further analyzed by two co-investigators during a two-hour synthesis workshop in September 2020. During this process, the study team extracted quotations that illustrated key themes. Consistent with prior analytic approaches to these data [17] and to provide a sense of the predominance of some themes relative to others, we documented the general number of transgender women who discussed each theme. We then used a gradient of qualitative markers (“*”) to indicate which themes were discussed by the majority or minority of participants by study site. In our next step, a subset of illustrative quotations was selected for presentation in this manuscript and labeled by age and employment status (e.g., full-time (FT), part-time (PT), or unemployed). Given the small sample drawn from a relatively small community and potential to identify individuals, we excluded race, HIV status, and site from quotation labels to safeguard participants’ anonymity. To enhance rigor, a peer-debriefing session was held with the interviewers, coders, and co-investigators. During this session, consensus was reached on all themes.

### Ethics

This study obtained ethical approval from the Virginia Commonwealth University (VCU) Office of Research and Innovation Institutional Review Board (IRB) (#HM20011245) and the University of Missouri – St. Louis (UMSL) IRB (#1179371–2). Informed consent was obtained from all subjects, or, if subjects were under 18, from a parent and/or legal guardian.

## Results

### Sample characteristics

Participant characteristics are listed in Table 1. Of the 19 transgender women enrolled in the study, 11 were from Richmond and 8 were from St. Louis. The average age of participants was 26.3 years with a range from 18 to 50 years. Most participants were racial minorities (*n* = 14), including 12 African-American, 1 Latina, and Asian-American transgender women. The remaining 5 participants identified as White. Four participants had only a primary school education and 8 had completed high school or the equivalent. Five participants had some college education and 2 had a college degree. Two participants reported being previously, but not currently, economically vulnerable. Approximately one-third (*n* = 6) of participants were unemployed, and among those employed (*n* = 13), most (*n* = 10) were under-employed. Fourteen participants reported housing insecurity in the prior year. Thirty-seven percent (*n* = 7) of participants reported being HIV-positive and 2 did not know their HIV status.

**Table 1:** Sample demographic and economic characteristics by site and total

	Site		Total
	Richmond	St. Louis	
Number of enrollees	11	8	19
Percentage of total sample	58%	42%	100%
*Demographic characteristics*			
Mean age in years	24.8	28.3	26.3
Age range (min, max)	20–30	18–50	18–50
Highest level of education			
Primary	0	50% (*n* = 4)	21% (*n* = 4)
High school diploma	64% (*n* = 7)	12% (*n* = 1)	42% (*n* = 8)
Some college	36% (*n* = 4)	12% (*n* = 1)	26% (*n* = 5)
College graduate	0	25% (*n* = 2)	11% (*n* = 2)
Race/Ethnicity			
African-American	73% (*n* = 8)	50% (*n* = 4)	63% (*n* = 12)
Latino/Hispanic	9% (*n* = 1)	0	5% (*n* = 1)
Asian	0	12% (*n* = 1)	5% (*n* = 1)
White	18% (*n* = 2)	38% (*n* = 3)	26% (*n* = 5)
*Economic characteristics*			
Economic vulnerability			
Previously	9% (*n* = 1)	12% (*n* = 1)	11% (*n* = 2)
Currently	91% (*n* = 10)	88% (*n* = 7)	89% (*n* = 17)
Currently employed			
Yes	64% (*n* = 7)	75% (*n* = 6)	68% (*n* = 13)
No	36% (*n* = 4)	25% (*n* = 2)	32% (*n* = 6)
Full-time employed^a^			
Yes	43% (*n* = 3)	0	23% (*n* = 3)
No	57% (*n* = 4)	100% (*n* = 6)	77% (*n* = 10)
Lacked housing in past year			
Yes	73% (*n* = 8)	75% (*n* = 6)	74% (*n* = 14)
No	27% (*n* = 3)	25% (*n* = 2)	26% (*n* = 5)
*HIV Characteristics*			
HIV Status			
Positive	27% (*n* = 3)	50% (*n* = 4)	37% (*n* = 7)
Negative	64% (n = 7)	38% (*n* = 3)	53% (*n* = 10)
Unknown	9% (*n* = 1)	12% (*n* = 1)	11% (*n* = 2)
*Gender Transition*			
Initiated hormone therapy			
Yes	82% (*n* = 9)	75% (*n* = 6)	79% (*n* = 15)
No	18% (*n* = 2)	25% (*n* = 2)	21% (*n* = 4)
Legal gender marker change			
Yes	45% (*n* = 5)	38% (*n* = 3)	42% (*n* = 8)
No	55% (*n* = 6)	62% (*n* = 5)	58% (*n* = 11)
Legal name change			
Yes	55% (*n* = 6)	25% (*n* = 2)	42% (*n* = 8)
No	45% (*n* = 5)	75% (*n* = 6)	58% (*n* = 11)

^a^ Excludes transgender women who were unemployed

### Emergent themes

One in-depth interview was conducted with each of 19 transgender women for a total of 19 interviews. Table 2 lists the emergent themes from these interviews within each of the six previously-identified study topics. Those topics and emergent themes were: (i) linkage to supportive economic services (4 themes), (ii) employment readiness training (3 themes), (iii) personal finance education (2 themes), (iv) gender transition supports (3 themes), (v) HIV prevention and care (3 themes), and (vi) intervention preferences and concerns (10 themes). The relative frequency with which participants discussed each theme is also provided in Table 2 as a proxy for the overall level of enthusiasm for each approach. A summary of each theme with illustrative quotations is below.

**Table 2:** Summary emergent themes and relative frequency of discussion by study topic

Study topic	Relative frequency of discussion^**a**^		Emergent themes
	Richmond	St. Louis	
**Linkage to Supportive Economic Services**	****	****	Provide vouchers to address insufficient funds
	***	**	Provide support to communicate and negotiate with banking institutions
	****	*	Trans-friendly awareness training, assessment, and support to employers/employees
	*		Database of trans-friendly employers and jobs
**Employment Readiness Training**	***	*	Guidance to improve resume or CV
	*	*	Guidance to improve job application and interview skills
	****	***	Support for job/career planning and sustainment through hardship
**Personal Finance Education**	****	****	Assistance with how to budget and save
	***		Accessing and improving credit
**Gender Transition Supports**	****	**	Support for achieving feminine appearance using non-medical services or products
	****	*	Support for obtaining clinical/medical gender transition services
	**		Educate and avoid dangerous, unskilled transition services
**HIV Prevention and Care**	****		Improve authentic HIV counseling and testing
	**		Offer other HIV-related harm reduction services
	*		Not too much attention/focus on HIV given its stigma associated with transgender women
**Intervention Preference and Concerns**	*	*	Concerns regarding participant misuse or ineligibility of resources
	**		Concern about transportation to participate in intervention
	****	*	Preference for visibly-trans intervention for all transgender women
	*	*	Important for safe zone, network, and locale
	****		Enabling group empowerment by sharing and learning from other transgender women
	**		Addition of support for responding to gender-based violence
	****	***	Best modes of advertising the intervention (flyer, social media, etc.)
	****	****	Best places to advertise the intervention (clinic, transgender women friendly club, etc.)
	***	****	Duration of intervention (responses to the 4-h length and/or 12 weeks)
	***	****	Preference for intervention group size

^a^ Relative frequency of responses are denoted by: * discussed by < 20% transgender women; ** discussed by 20 to 35% transgender women; ****discussed by 35 to 50% transgender women; ****** discussed by > 50% of transgender women

#### Linkage to supportive economic services

##### Provide vouchers to address insufficient funds

Most participants were enthusiastic about the idea of vouchers. They described multiple ways in which vouchers could support their legal and physical gender affirmation. As one participant stated, it was important to minimize financial barriers for all transgender women. However, they also cautioned against proscriptive limits on how the vouchers could be used.

“You mean, have these girls get their name changed, and all of that, everybody don’t have the funds, so we have to find a way to have these organizations that help reach out for help with that particular.” – 50 years old, employed part-time.

“I know a lot of trans women who, like, the main thing that’s stopping them from getting on estrogen is financial limitations.” – 21 years old, employed part-time.

“Supporting on an individual basis what that person’s transition may need is gonna be really important.” – 18 years old, employed part-time.

##### Provide support to communicate and negotiate with banking institutions

Participants described multiple barriers to engaging with banks. Lack of trust in governmental institutions, the humiliation of having to use a legal name on official documents that may not match their identity, and inexperience with banking or bank accounts were cited. Thus, an intervention that provided support for engaging with banking institution was regarded positively.

I would definitely say the most trans women I know [are not] comfortable enough to really go into like, places like employment or banking or even finding shelter when it comes to places that might deny them because of who they are because certain people don’t really accept them in that space.” – 18 years old, employed part-time.

“You know, so to help us build a resume and, you know, help us find banking**,** that’s a wonderful thing that we should know and we should have.” – 25 years old, unemployed.

##### Trans-friendly awareness training, assessment, and support to employers/employees

Multiple participants raised the importance of ensuring employers and co-workers are prepared to treat transgender employees with respect. Suggestions ranged from sensitivity training for the staff to “secret shopper” approaches to test how transgender job applicants are treated. However, one participant expressed concern that incentivizing employers to hire transgender women would allow them to benefit from underpaying transgender employees.

“Making sure that they are absolutely ready with no ifs, ands, or buts about it. So it’s like if the situation occurs you know that you’ve trained them to your best abilities.” – 23 years old, unemployed.

“I’m never 100% sure about which places that I go to are accepting of trans women or not, so it’d be good to have something that can, like, find out information on that.” – 20 years old, employed part-time.

“I think that the only problem is that giving the company a financial reason to hire a trans woman is kind of, in a way, kind of benefitting off, oh my god we have a trans woman and we don’t have to pay her all the way. Ya know, that’s like one of those things I feel like that’s a problem.” – 18 years old, employed part-time.

##### Database of trans-friendly employers and jobs

Suggestions to create a database of employers and employment opportunities that welcome transgender employees emerged spontaneously. Such a database would prevent transgender women from wasting their time applying for jobs where they are unlikely to be hired and prevent the psychological stress of anticipating rejection.

“If you had, like, maybe a website or something that people can access. And even if, like, they aren’t able to get involved with the internship program, they can have, like, a list of businesses that you know are, like, won’t turn away transgender women for applying.” – 20 years old, employed part-time.

“So that was an organization that would literally just find jobs and be like, we are trying to get this trans woman hired. Do you have openings? It is also like really like would be a huge burden lifted off a lot of people.” – 26 years old, employed full-time.

#### Employment readiness training

##### Guidance to improve resume

Some participants saw value in obtaining support with resume/CV writing. Suggestions included assistance with grammar and marketing their skill sets, especially in light of low educational attainment of many economically vulnerable transgender women. As one transgender women stated, even minor edits or application reviews could be instrumental in improving employability.

“Like, it took me years to even read - well, I guess to write a resume or know how to write one and it’s kinda like, [sighs] I wish I woulda knew all this earlier.” – 30 years old, employed full-time.

“I feel like there are a lot of transgender women who would drop out of high school, and sometimes they don’t know what to do. Like how to fill out job applications and how to do a correct resume and sometimes what’s maybe stopping them from getting that job. It’s just, you know, their resume is incorrect or it’s not punctual and you know, the punctuation is not corrected. Things like that are important when you’re looking for a job because if you can’t spell and you can’t type or you know, write out a complete sentence and you can’t spell things correctly, that’s like an issue.” – 21 years old, employed part-time.

##### Guidance to improve job application and interview skills

In addition to support with resume writing, a few participants described the benefits of training on communication and interviewing skills. Assistance in navigating employment discussions as a transgender woman was valued.

“Life skills, making sure people have that experience of interviews and how to present yourself.” – 18 years old, unemployed.

“It could pretty much help on a lot a. I would say some people don’t really have, like, appropriate communication skills.” – 30 years old, employed full-time.

##### Support for job/career planning and sustainment through hardship

Participants wanted help finding stable employment and navigating discrimination they would encounter. An overall sentiment was that the experience of stigma and transphobia among participants resulted in high job instability, making it difficult to access higher paying jobs or save money between paychecks. Transgender women wanted more support not only in applying for better jobs, but also in managing having one’s “feelings hurt” by recurring job denials or losses.

“It’s very unstable. It’s basically paycheck to paycheck to paycheck or job to job to job …” – 18 years old, employed part-time.

“Finding jobs that wouldn’t be discriminatory because half the time it’s hard applying for a job because you’re like, okay, well here’s a chance of getting my feelings hurt and not getting the job and finding out these people are horrible.” – 26 years old, employed full-time.

#### Personal finance education

##### Assistance with how to budget and save

Participants were positive about the idea of learning to budget and save money, often because the jobs available to them were low wage, part-time, and/or temporary. Several participants also spoke to the additional expenses borne by transgender women who needed to spend additional money to maintain their gender presentation.

“It’s basically paycheck to paycheck to paycheck or job to job to job and so there’s not much time to sit and think. What am I going to do with this money?” – 18 years old, employed part-time.

“I didn’t know about saving for the future, like you don’t think about all that stuff you just...the world is going on now so you that’s all re thinking about, but you truly have to think about the future and the choices that you do make now will affect the future.” – 26 years old, unemployed.

##### Accessing and improving credit

A history of working outside the formal job sector often meant that transgender women did not have credit or that they had poor credit ratings from unpaid debt. A few participants spoke to the benefits of assistance with accessing and improving credit.

“Yeah classes for like to help you get your credit back or stuff like that.” – 27 years old, employed part-time.

“Right! So yeah that’s like, you know, people already when they’re younger they mess up their credit, so that’s wonderful, I like that.” – 26 years old, unemployed.

#### Gender transition supports

##### Support for achieving feminine appearance through non-medical supports

Nearly every participant described the value of support for their gender presentation, ranging from makeup classes to hair removal. This theme often occurred in the context of discussions about vouchers for transgender women.

“Teach them how to wear it. You know? I think that would go a long way. ‘Cause the sexiest option isn’t always the prettiest option it’s just not and it’s often not. But when you weren’t raised as a woman you didn’t have that socialization as a woman you don’t know and you’re trying to figure it out and it’s hard.” – 27 years old, employed part-time.

“I think that’s good because like I said, somebody that wants to transition, if they don’t have those, they might you know feel really bad and not even feel like they’re meant to transition, but that little bit of help...just putting a wig on somebody that’s nice or whatever the case might be...is gonna give them that boost of confidence and feel like they can go like up to the world and feel more real or whatever the case might be-more confident.” – 26 years old, unemployed.

##### Support for obtaining clinical/medical gender transition services

Many participants also described the high cost of gender affirming medical services and expressed a desire to use a grant or voucher program to support access to these services.

“You know, the most important things that people are trying to work towards getting done is surgery and hormones.” – 23 years old, employed full-time.

“If you don’t have a family support, or you don’t have a job, or you don’t have no one you can, you know, rely on that can help, you know, anymore, cause laser hair removal, surgeries - it ranges me, you know, for my face alone, $300 per session, and I have 3 sessions so far. So it can, you know, in a month you would spend, like, $1,000 to $2,000 in a month.” – 25 years old, unemployed.

##### Educate about and avoid dangerous, unskilled transition services

Financial barriers to accessing gender affirming medical care within the formal healthcare sector sometimes led participants to seek lower cost, unskilled services outside of the formal sector.

“And, a lot of us don’t have the funding for that. That’s why I stopped going, cause I don’t have the money for that. And, I was going to kind of like a bootleg one, and I got kind of like a burn on my face. I don’t know if you can see it? It’s kind of like a dark spot?” – 24 years old, unemployed.

“Some of them-I have friends that are dead because they have shot the liquid that pure silicone into their chest or into their face and into their body and its traveled into their heart. Because they wanna be ladies.” – 50 years old, employed part-time.

#### HIV prevention and care

##### Improve authentic HIV counseling and testing

Several participants supported inclusion of HIV testing as part of a community-based intervention. As described below by two transgender women, this was seen as an important intervention component given that some participants feared being judged by health providers who stigmatized transgender persons, which reduced care-seeking for testing services for HIV or other sexually transmitted infections. Participants felt that having more visibly transgender-friendly HIV health providers via mobile clinics or “trucks”, for example, would enable more transgender women to determine their HIV status and that of their sexual partners.

“So I’m able to see a doctor and get tested and stuff like that. If I wouldn’t been getting hormones from a doctor I wouldn’t have gotten tested and I would have not known my status. You get what I’m saying? So, that is like this wonderful just like random test and stuff like that. And like you said, just more accessibility for that, so that’s wonderful. .. You’re not going to the doctors frequently, but something like that if there’s a truck out or whatever you know just doing like whatever but you know just seeing and being like, “Oh let’s get tested”, like you know what I’m saying? So yeah, it being more in the community would help way better. And some people don’t feel comfortable going to the doctors you know?” – 26 years old, unemployed.

“The reason why I agree with that is because, you know, most people you know... they hold off, you know, the HIV testing. Um, and I feel like that’s not okay. A lot of people are scared, you know, to talk to their doctor about getting a HIV test cause they don’t want the doctor to, you know, look at them or judge them. Because, you know, you do have some doctors that will-will do that.” – 25 years old, unemployed.

##### Offer other HIV-related harm reduction services

In addition to HIV testing, participants described the importance of harm reduction services as part of any HIV intervention. This included provision of a safe needle exchange program or distribution of condoms.

“Um, perhaps like an access to fresh needles because being an addict, its really hard, a lot of people are addicts and that’s a really easy way for people to spread HIV is by sharing needles.”– 26 years old, employed full-time.

“Yeah. Need tons of that, actually. I like that, too. Would that include, like, as far as, like... preventative measures as far as, like, condoms and all that kind of stuff too.” – 28 years old, employed part-time.

##### Not too much attention/focus on HIV given its stigma associated with transgender women

While some participants were supportive of including HIV components in an intervention, they felt it should only be one component nor the main topic. Having too much focus on HIV prevention in an intervention targeting transgender women was viewed as off-putting, ultimately discouraging program participation.

“But, I just feel like you don’t want to make it too much about HIV, because then it might scare trans women … Ya know, if that’s all they talk about ‘cause, ya know, a lot of people don’t even like to go get tested. Sometimes seeing HIV on the front of something is like, ‘Ooo, do I really want to go to this thing, and they talking about HIV the whole time?’” – 21 years old, employed part-time.

#### Intervention preference and concerns

##### Concerns regarding participant misuse or ineligibility of resources

A few participants expressed concerns about potential misuse of economic support. For example, as one transgender woman indicated, there would likely be a need to specify any conditions associated with receipt or use of study-provided resources. There was a sentiment among some participants, as described below, that provision of resources should be targeted to those most in need or who meet a set of eligibility criteria – such as those who were unemployed, underemployed, or unable to work.

“I don’t want it to become another one of those things where people use and abuse it. Like, if they use it make sure they use it for the right reasons.” – 23 years old, unemployed.

“And what I mean by work for what we want, I basically mean because a person can lay on they tail and-and receive these, you know, these funds, and they able to work. And why then they not, you know, doing it? I feel like if you want to receive the funds, you should be putting in effort to show why you need the funds.” – 25 years old, unemployed.

##### Concern about transportation to participate in intervention

Participants raised concerns about the ability to get to an intervention due to lack of transportation and the risk of violence when using public transportation.

“Because they were having difficulties with rides or, um, some people get stuck at work and so the person that you normally ride with from that side of town is leaving straight from work so then they don’t have a ride.” – 23 years old, unemployed.

“Like bus rides sometimes be...I would say harassment especially to be like at a bus stop and somebody’s like hitting on you and then you, like, are bashed for not answering or bashed for disclosing the fact that you’re trans. It can be like hit or miss. That is something to think about.” – 23 years old, unemployed.

##### Preference for visibly-trans intervention for all transgender women

Participants highlighted the importance of making sure that transgender women knew the intervention was specifically for them by ensuring visibility of transgender women in the advertising.

“As long as you, like, get like, different kinds of trans people. Like, don’t have, like, five White trans girls who are, like, in their 20’s or like, are older and have already completely transitioned or whatever. Make sure there’s, like uh, voices for different transgender women of color. As long as you’re able to take in different transgender women’s perspectives on stuff then that could be really helpful.” – 20 years old, employed part-time.

“Yeah, for me. It’s easier to identify with when you can see, like, “trans.” And then the big picture is to put trans in front, like if you put your trans in front of it or if you put incentive and then trans people are going to show up. So that’s definitely something. I would put in bold trans, like if, if you guys, you know, because sometimes like I said, it’ll be like minority health thing and it has and it has these topics, but if you make the main topic trends more people are going to be like okay, so this is actually for us or this is mostly about us so it’ll make them come out.” – 21 years old, employed part-time.

##### Important for safe zone, network, and locale

Several participants described the importance of ensuring that any intervention would take place somewhere that felt safe to transgender women. For example, one transgender woman stated that clubs that were frequented by transgender women were perceived as existing “safe zones” and would be readily identified as a safe and fun venue for study activities. Another participant suggested that the study team should pre-specify which venues they hoped to target as a means of ensuring that potential participants would feel welcomed and safe. We also found that safety was conceptualized as having confidence in the study through community referrals. As one transgender woman described, accessing a diverse transgender network could best ensure safe participation.

“But, really the clubs. Only because like I said they already in that safe zone. So, to hear it being talked about you be like ‘ok, well if we’re already in a safe place they’re about another safe place.’ So it’s like a ‘ok,’ and it kinda rings a bell ‘cause you’re going out to have fun.” – 23 years old, unemployed.

“The different places that you’re looking forward to working with would be good. ‘Cause that’ll also then show ‘ok well, girl, if they go … this flyer in here – they comfortable with having us in here.’”– 23 years old, unemployed.

“I would say the best ways to get more, like, personal referrals from other women or just like other people in the LGBT community that they know who are more headstrong, people who are more educated or actually want to be a part of something like that, something to help support transwomen in their own community I would say.” – 18 years old, unemployed.

##### Enabling group empowerment by sharing and learning from other transgender women

Almost every participant mentioned the importance of having someone who reflected their lived experience to lead any intervention. They spoke specifically of gender identity and race as important, not only to reduce concerns of inadvertent stigmatizing behaviors but also to serve as role models.

“… finding someone who’s a trans woman who has made it, one of the main people, [Name redacted] is one of those people who’s just really got her shit together and it’s awesome. It’s just pulling people from different organizations could be a good thing as well.” – 26 years old, employed full-time.

“I feel like that is a great setup, cause, you know, when you have a small group of different transgender women on different walks of life, you know, you can-you can learn more cause each person has a different story. My story may not be the same as that person or that person, you know, so it’s more like, you know, you getting to know different people as well as you hearing different, you know, stories from different people. So it’s more of, you know, you gaining a, you know, bond.” – 25 years old, unemployed.

##### Addition of support for responding to gender-based violence

In addition to economic support, participants discussed the need for interventions to address gender-based violence experienced by transgender women. Examples included a self defense class, guidance about whether or how to carry protective supplies (i.e., mace), and safety planning for contexts where transgender women felt threatened or in danger.

“Uh, self-defense class of some kind, ‘cause regardless of where you are, being openly trans is definitely a risk for hate crimes and stuff, so I’m sure that learning how to properly defend yourself would be able to help a lot of people.” – 20 years old, employed part-time.

“I wouldn’t probably need protecting from anything like that, other than maybe, I guess, carrying mace or something like that. Maybe a gun, but other than that I would say I wouldn’t have to be that worried, but I would say for other fellow sisters I would definitely say they would definitely need protection of somewhat when it comes to situations where they feel they are in danger or something.” – 18 years old, unemployed.

##### Best modes of advertising the intervention

Participants suggested multiple modes for promoting a future intervention. Transgender-specific social media, pamphlets, and word of mouth were commonly mentioned.

“Um, flyers ‘cause you could use them everywhere, social media, in the clubs, here, like just different places that people go to. And the hair stores ‘cause they famous for going there.” – 23 years old, unemployed.

##### Best places to advertise the intervention

Participants encouraged advertising the intervention through existing transgender community organizations and networks.

“Community outreach. I’m not. .. door to door but I guess not door to door but I feel like maybe like flyers, you guys trying to hand out flyers, like just um, like a community center for Trans would be very helpful to specifically trans.” – 21 years old, employed part-time.

“Somebody that they know. You need people like me to tell the girls, girl I did it already and it’s cool. You need to do it because I already did.” - 34 years old, unemployed.

“You guys have to go face to face with these people. With this group here, they’re not going to come to you. You gotta go to them. Because they have been so battered they don’t trust people. Talking to trans organization and trans leaders in the St. Louis area who are already involved with helping trans-women because they already know what’s going on.” – 27 years old, employed part-time.

##### Length and duration of intervention

Most participants responded positively to the idea of a 48-h intervention that lasted 4 hours and took place weekly for 12 weeks, especially if sessions took place in the evening. However, a few participants noted that length and duration of the intervention may be too much for some women.

“I would say ‘cause it flies by. Like, how long we been sitting here for almost two and it don’t even feel like it.” – 23 years old, unemployed.

“It’s definitely going to have to be evening. It would have to be my five to like five to seven, something like that.” – 27 years old, employed part-time.

“I feel like it might be kinda hard to find consistency when it comes to certain people and everything. It’s either they’re too busy or even they might just back out from the full time or something.” – 18 years old, unemployed.

##### Preference for intervention group size

Participants were asked about the acceptability of a group size of 5–8 participants. All agreed that number of participants would be ideal, with most preferring the smaller size.

“You don’t want too many people ‘cause you want it to be, you know, like one on one kind of experience even though it’s a group but you wanna feel everybody’s energy and you want everybody to feel- you don’t want it to be too many personalities ‘cause they on hormones.” – 27 years old, employed part-time.

## Discussion

This study sought to identify and explore the intervention preferences of economically vulnerable transgender women to inform the design of future structural HIV prevention interventions. Participants expressed the strongest support for vouchers, particularly if use of the funds were unrestricted in order to be applied for high priority expenses such as gender affirmation. While U.S.-based research on cash transfers (e.g. grants and vouchers) for HIV prevention is scant [31], data from low-income countries indicate these interventions may reduce HIV risk behaviors and incident infections, particularly among the most economically vulnerable [31–33]. Gender affirmation is a priority for transgender women, and unmet needs for gender affirmation are known to increase HIV risk behavior [17, 34]. Microeconomic interventions for transgender women should include financial support for gender affirming interventions (e.g., makeup, hormones, legal name change) to promote efficacy as well as acceptability [17].

Pervasive stigma and discrimination against transgender women is associated with high unemployment and/or low wage jobs [16]. Study participants were very supportive of job readiness training, yet it was obvious that readiness alone would not ensure employment. Stigma reduction interventions for employers are necessary to ensure not only that they hire transgender women, but also that the transgender women they hire are safe from harassment and discrimination in the workplace.

Chronic under- and unemployment and survival via the informal economy means that many economically vulnerable transgender people do not have the opportunity to learn how to budget, save, or interact with financial institutions. Study participants expressed a strong desire to learn these financial literacy skills. Microeconomic interventions with other populations have identified financial literacy as a core component of economic and gender empowerment [35, 36], and our data also underscore the potential importance of including financial training in microeconomic interventions geared towards vulnerable transgender women.

While there was some support for integrating HIV prevention within a microeconomic intervention, transgender women enrolled in the study were clear that HIV prevention (as well as care and treatment) should not be the intervention’s focus. Reasons for minimizing the emphasis on HIV stemmed from feeling targeted and stigmatized by public health initiatives as being associated with and drivers of the HIV epidemic. In fact, existing literature supports this non-stigmatized approach to HIV prevention. Integrated health education and microeconomic interventions are increasingly viewed as more effective in reducing HIV risk than interventions focused solely on risk reduction education [37, 38]. While the efficacy of microeconomic interventions can be influenced by existing stigma, poverty, and gender inequality, microeconomic interventions that are integrated with health behavioral activities may build more resilience to these factors through financial empowerment, increased self-esteem, social support, and solidarity, in addition to improved mental health and self-efficacy [39]. In turn, these intermediate outcomes may reduce engagement in HIV risk behaviors [29, 40].

When implementing a microeconomic intervention, participants noted the importance of working in small groups and ensuring that economic and behavioral education centers around the unique needs of transgender women. Interventions should promote visibility and empowerment of transgender women and be advertised through existing transgender networks. Safe transportation to the intervention site should be ensured, and groups should be relatively small and take place in spaces where transgender women feel comfortable. Other issues of importance to transgender women such as gender-based violence and harm reduction should be addressed [30, 41].

### Limitations

The results of this study should be interpreted in light of its limitations. Given the cross-sectional design and small sample size, we were unable to reach saturation by sub-group, such as city or HIV status. Future studies may benefit from a larger sample size in each city. Social desirability bias may have influenced how participants responded to questions, especially when participants were asked to agree or disagree with proposed intervention length, duration, or size. While the RAs who conducted the interviews underwent training in qualitative science, community awareness, and ethics in human subject research, they did not always reflect the racial background, economic status, or gender identities of the study participants. These differences likely impacted the amount and types of information transgender participants were willing to share during the interviews. Future studies are likely to benefit from increased diversity among interviewers. Nonetheless, even with these limitations, this study provides important and previously absent data relating to transgender women’s preferences and priorities for microeconomic interventions to reduce HIV vulnerability. Findings can be used to inform future formative and intervention design research.

## Conclusion

Flexible microeconomic interventions that support gender affirming interventions, improve financial literacy, and provide living-wage non-stigmatizing employment are desired by economically vulnerable transgender women. While not focused on HIV, such interventions have the potential to reduce the structural drivers of HIV vulnerability among transgender women.

## Data Availability

The datasets generated and analysed during the current study are not publicly available due risk of breaching participant confidentiality based on the small sample size and enrollment of a very specific vulnerable population.

## Abbreviations

CAB: Community advisory board
CBO: Community-based organization
FT: Full-time employed
HIV: Human immunodeficiency virus
IRB: Institutional review board
LGBTQ: Lesbian, gay, bisexual, transgender, queer and/or questioning
MO: Missouri
MSM: Men who have sex with men
PrEP: Pre-exposure prophylaxis
PT: Part-time employed
RA: Research assistant
STI: Sexually transmitted infection
TW: Transgender women
UMSL: University of Missouri – St. Louis
US: United States
VA: Virginia
VCU: Virginia Commonwealth University
